# Deep learning-based decision support system for the diagnosis of neoplastic gallbladder polyps on ultrasonography: Preliminary results

**DOI:** 10.1038/s41598-020-64205-y

**Published:** 2020-05-07

**Authors:** Younbeom Jeong, Jung Hoon Kim, Hee-Dong Chae, Sae-Jin Park, Jae Seok Bae, Ijin Joo, Joon Koo Han

**Affiliations:** 1 Department of Radiology, Seoul National University Hospital, 101 Daehak-ro, Jongno-gu, Seoul, 03080 Korea; 2 Department of Radiology, Seoul National University College of Medicine, 103 Daehak-ro, Jongno-gu, Seoul, 03080 Korea; 3 Institute of Radiation Medicine, Seoul National University Medical Research Center, 103 Daehak-ro, Jongno-gu, Seoul, 03080 Republic of Korea

**Keywords:** Gall bladder cancer, Gall bladder

## Abstract

Ultrasonography (US) has been considered image of choice for gallbladder (GB) polyp, however, it had limitations in differentiating between nonneoplastic polyps and neoplastic polyps. We developed and investigated the usefulness of a deep learning-based decision support system (DL-DSS) for the differential diagnosis of GB polyps on US. We retrospectively collected 535 patients, and they were divided into the development dataset (n = 437) and test dataset (n = 98). The binary classification convolutional neural network model was developed by transfer learning. Using the test dataset, three radiologists with different experience levels retrospectively graded the possibility of a neoplastic polyp using a 5-point confidence scale. The reviewers were requested to re-evaluate their grades using the DL-DSS assistant. The areas under the curve (AUCs) of three reviewers were 0.94, 0.78, and 0.87. The DL-DSS alone showed an AUC of 0.92. With the DL-DSS assistant, the AUCs of the reviewer’s improved to 0.95, 0.91, and 0.91. Also, the specificity of the reviewers was improved (65.1–85.7 to 71.4–93.7). The intraclass correlation coefficient (ICC) improved from 0.87 to 0.93. In conclusion, DL-DSS could be used as an assistant tool to decrease the gap between reviewers and to reduce the false positive rate.

## Introduction

Gallbladder (GB) polyps are commonly detected during ultrasonography (US), with a reported prevalence that ranges from 0.3 to 9.5%, and they present a tricky clinical question regarding their malignancy^1–9^. They can be divided into two groups: benign nonneoplastic and neoplastic polyps, which include adenomas and adenocarcinomas^9–11^. As adenomatous polyps can be malignant or can become malignant, it is important to differentiate and properly manage an adenomatous polyp^9^.

US has been considered image of choice for GB polyp. To diagnose neoplastic GB polyps, US showed an 80% accuracy^12^. Although US provided relatively good performance for diagnosing GB polyps, it had limitations in differentiating between nonneoplastic polyps and neoplastic polyps.

Recently, deep learning has been applied in various fields^13^. The deep learning models using image input usually have been developed to classify lesion or nonlesion, to group the lesion type, to detect the lesion, or to segment the lesion^14^. Additionally, the US field has less deep learning studies than other modalities. Most of the topics have been focused on lesion classification for breast, liver, and thyroid, and other minor topics include automatic carotid ultrasound image analysis, myositis type classification, and spine level identification^15^.

Several studies have shown that deep learning could enhance the performance of the radiologist^13^. A deep learning model could help the radiologist in detecting the pulmonary nodule on a chest radiograph, interpreting the knee magnetic resonance imaging (MRI), and detecting a cerebral aneurysm on magnetic resonance angiography^16–18^. To our knowledge, no articles have been published applying the deep learning method to differentiate GB polyps on US. The purpose of our study is to determine if a deep learning-based decision support system (DL-DSS) is helpful for the differential diagnosis of neoplastic GB polyps on US.

## Results

### Patient population

A total of 535 patients were included in our study group, including 357 patients with nonneoplastic polyps and 179 patients with neoplastic polyps. We divided selected patients into two groups to make two temporally independent datasets: the development dataset (n = 437) and test dataset (n = 98).

The pathologic diagnoses of GB polyps were as follows: nonneoplastic polyp (n = 357), including cholesterol polyp (n = 349); hyperplastic polyp (n = 1); inflammatory polyp (n = 7); neoplastic polyp (n = 179), including adenoma (n = 95); intracystic papillary neoplasm (n = 1); intracystic tubulopapillary neoplasm (n = 8); fibroepithelial polyp (n = 1); adenocarcinoma (n = 62); intracystic papillary neoplasm with an associated invasive carcinoma (n = 10); papillary carcinoma (n = 1); and adenosquamous carcinoma (n = 1).

From the 535 patients, we collected total of 6,056 cropped polyp images: 3,629 images of nonneoplastic polyps and 2,427 images of neoplastic polyps. The development dataset consisted of 3,200 images of nonneoplastic polyps and 1,971 images of neoplastic polyps. The test dataset consisted of 429 images of nonneoplastic polyps and 456 images of neoplastic polyps.

### Baseline characteristics of data sets

There was no significant difference in the population characteristics (age, man to woman ratio, proportion of nonneoplastic and neoplastic polyps, and size of polyps) between the development set and test set (p ≥ 0.05). The baseline characteristics of the data sets are described in Table 1.

**Table 1:** Baseline Characteristics of Data Sets.

Characteristics	Development Set	Test Set	Total	p
No. of Patients	437	98	535	
No. of Images	5,171	885	6,056	
Age (y)	52.2 ± 13.5 (21–53–87)	55.2 ± 12.5 (26–55–86)	52.7 ± 13.4 (21–54–87)	0.05
No. of Man Patients	198 [45.3]	37 [37.8]	235 [43.9]	0.17
No. of Patients with Nonneoplastic polyp	294 [67.3]	63 [64.3]	357 [66.7]	0.6
No. of Patients with Neoplastic polyp	144 [33.0]	35 [39.3]	179 [33.5]	
Size of Polyp	12.2 ± 7.1 (4.1–10.4–47.2)	13.4 ± 6.9 (4–12.3–35)	12.4 ± 7.0 (4–10.6–47.2)	0.14
Size of Nonneoplastic Polyp	9.4 ± 3.6 (4.1–8.9–21.4)	9.9 ± 2.9 (4–9.9–15.4)	9.4 ± 3.5 (4–9.1–21.4)	0.27
Size of Neoplastic Polyp	18.1 ± 8.6 (4.3–16.0–47.2)	19.8 ± 7.5 (4.6–17.7–35)	18.4 ± 8.4 (4.3–16.4–47.2)	0.29
**Development Set**	**Nonneoplastic polyp**		**Neoplastic polyp**	
No. of Patients	294		144	
Age (y)	48.5 ± 12.5		59.7 ± 12.4	<0.001
No. of Man Patients	124 [42.2]		74 [51.4]	0.07
Size of Polyp	9.4 ± 3.6		18.1 ± 8.6	<0.001
**Test Set**	**Nonneoplastic polyp**		**Neoplastic polyp**	
No. of Patients	63		35	
Age (y)	51.1 ± 10.9		62.5 ± 11.9	<0.001
No. of Man Patients	17 [27.0]		20 [57.1]	0.07
Size of Polyp	9.9 ± 2.9		19.8 ± 7.5	<0.001

Note.- Size of polyp was calculated from the one maximum value from each patients. Data in parentheses are minimum, median, maximum values, respectively. Data in brackets are percentage.

**Figure 1:**
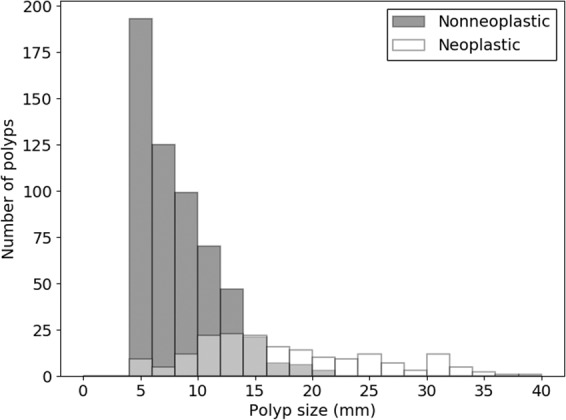
Size distribution histogram of the polyps in the whole dataset. The average size of all polyps was 12.4 mm, and the average size of nonneoplastic and neoplastic polyp was 9.4 mm and 18.4 mm respectively. There was a substantial overlap zone between nonneoplastic polyps and neoplastic polyps.

In the both development set and test set, patient age and polyp size showed statistically significant differences between nonneoplastic and neoplastic polyp (p < .001). The average size of nonneoplastic and neoplastic polyps was 9.4 ± 3.5 mm and 18.4 ± 8.4 mm, respectively. The optimal size cutoff for differentiating a neoplastic polyp was over 13.1 mm (with sensitivity 70.1%, and specificity 87.1%) form the receiver operating characteristic (ROC) curve analysis. However, there was a substantial amount of size overlap between the nonneoplastic and neoplastic polyps. The size distribution histogram is shown on Fig. 1.

### Outcomes of clinical validation of DL-DSS

#### *Step 1: Diagnostic performance of image analysis by three human reviewers*

Table 2 summarizes the diagnostic performance of the three human reviewers. Three reviewers showed different area under the receiver operating characteristic curve (AUC) from 0.78 to 0.94. Reviewer A showed relatively good sensitivity (31 of 35 [88.6%]) and specificity (54 of 63 [85.7%]) on classifying the neoplastic polyp. However, reviewers B and C showed relatively low specificities of 43/63 (68.3%) and 41/63 (65.1%) respectively. The overall accuracy was between 68/98 (69.4%) and 85/98 (86.7%).

**Table 2:** Diagnostic Performance of Reviewers and DL-DSS.

	AUC	Comparison	Sensitivity	Specificity	Accuracy	F-1 Score
**Step 1: Initial Image Analysis**		Step 1 vs Step 2 (p value)				
Reviewer A	0.94 [0.88–0.98]	0.49	88.6 (31/35) [73.3–96.8]	85.7 (54/63) [74.6–93.3]	86.7 (85/98) [74.1–94.6]	0.827 [0.737–0.870]
Reviewer B	0.78 [0.68–0.85]	<0.01	71.4 (25/35) [53.7–85.4]	68.3 (43/63) [55.3–79.4]	69.4 (68/98) [54.7–81.5]	0.624 [0.509–0.704]
Reviewer C	0.87 [0.79–0.93]	0.11	97.1 (34/35) [85.1–99.9]	65.1 (41/63) [52.0–76.7]	76.5 (75/98) [63.8–85.0]	0.747 [0.687–0.760]
**Step 2: DL-DSS Alone**						
DL-DSS	0.92 [0.85–0.97]		74.3 (26/35) [56.7–87.5]	92.1 (58/63) [82.4–97.4]	85.7 (84/98) [73.2–93.9]	0.788 [0.663–0.867]
**Step 3: DL-DSS Aided**		Step 1 vs Step 3 (p value)				
Reviewer A	0.95 [0.88–0.98]	0.65	85.7 (30/35) [69.7–95.2]	93.7 (59/63) [84.5–98.2]	90.8 (89/98) [79.2–97.1]	0.869 [0.770–0.921]
Reviewer B	0.91 [0.83–0.96]	<0.01	80.0 (28/35) [63.1–91.6]	93.7 (59/63) [84.5–98.2]	88.8 (87/98) [76.9–95.8]	0.836 [0.723–0.902]
Reviewer C	0.91 [0.83–0.96]	0.17	91.4 (32/35) [76.9–98.2]	71.4 (45/63) [58.7–82.1]	78.6 (77/98) [65.2–87.9]	0.753 [0.673–0.787]

Note.- Data in brackets are 95% CI.

Table 3 summarizes the comparison with the US image findings between neoplastic polyps and nonneoplastic polyps. Among the three reviewers, the characteristic image findings of neoplastic polyps were single, larger, and sessile compared to the nonneoplastic polyps. A majority of the reviewers (two of three) reported lobulated surface contour, the presence of a vascular core, and heterogeneous internal echogenicity was also a common finding in the neoplastic polyps. Among the US findings, multiplicity, size, vascular core, and presence of gallstone showed good interobserver agreement (intraclass correlation coefficient (ICC) = 0.82–0.97). The shape, surface contour, and internal echo level showed moderate agreement (ICC = 0.65–0.79). From the multivariate logistic regression analysis, only the diameter of the polyp was a significant predictive finding of a neoplastic polyp. This was consistent result from all three reviewers (odds ratio (OR) 2.1, confidence interval (CI) 1.2–3.5, p < 0.01 for reviewer A, OR 1.5, CI 1.2–1.9, p < 0.001 for reviewer B, and OR 1.5, CI 1.1–2.2, p < 0.02 for reviewer C).

**Table 3:** Comparison of US findings.

Findings		Reviewer A			Reviewer B			Reviewer C		
		Nonneo	Neo	p	Nonneo	Neo	p	Nonneo	Neo	p
Multiplicity	Single	30.2% (19/63)	71.4% (25/35)	<0.001	38.1% (24/63)	74.3% (26/35)	<0.001	36.5% (23/63)	80.0% (28/35)	<0.001
	Multiple	69.8% (44/63)	28.6% (10/35)		63.9% (39/63)	25.7% (9/35)		63.5% (40/63)	20.0% (7/35)	
Size		9.4 ± 2.7	19.9 ± 7.5	<0.001	10.2 ± 2.9	19.5 ± 7.6	<0.001	9.6 ± 3.0	19.2 ± 7.9	<0.001
Shape	Pedunculated	95.2% (60/63)	57.1% (20/35)	<0.001	73.0% (46/63)	37.1% (13/35)	<0.01	81.0% (51/63)	40.0% (14/35)	<0.001
	Sessile	4.8% (3/63)	42.9% (15/35)		27.0% (17/63)	62.9% (22/35)		19.0% (12/63)	60.0% (21/35)	
Contour	Smooth	60.3% (38/63)	22.9% (8/35)	<0.001	19.0% (12/63)	8.6% (3/35)	0.24	60.3% (38/63)	20.0% (7/35)	<0.001
	Lobulated	39.7% (25/63)	77.1% (27/35)		81.0% (51/63)	91.4% (32/35)		39.7% (25/63)	80.0% (28/35)	
Gallstone	Absent	88.9% (56/63)	85.7% (30/35)	0.75	46.7% (21/45)	28.1% (9/32)	0.15	85.7% (54/63)	91.4% (32/35)	0.53
	Present	11.1% (7/63)	14.3% (5/35)		53.3% (24/45)	71.9% (23/32)		14.3% (9/63)	8.6% (3/35)	
Vascular Core	Absent	47.8% (22/46)	21.9% (7/32)	0.03	88.9% (56/63)	94.3% (33/35)	0.49	54.3% (25/46)	21.9% (7/32)	<0.01
	Present	52.2% (24/46)	78.1% (25/32)		11.1% (7/63)	5.7% (2/35)		45.7% (21/46)	78.1% (25/32)	
Internal Echo Level	Hypoechoic	41.3% (26/63)	62.9% (22/35)	0.03	64.5% (40/62)	51.4% (18/35)	0.30	34.9% (22/63)	57.1% (20/35)	0.05
	Isoechoic	58.7% (37/63)	34.3% (12/35)		33.9% (21/62)	42.9% (15/35)		61.9% (39/63)	40.0% (14/35)	
	Hyperechoic	0% (0/63)	2.9% (1/35)		1.6% (1/62)	5.7% (2/35)		3.2% (2/63)	2.9% (1/35)	
Internal Echo Pattern	Homogenous	66.7% (42/63)	68.6% (24/35)	1.00	71.4% (45/63)	42.9% (15/35)	<0.01	58.7% (37/63)	11.4% (4/35)	<0.001
	Heterogenous	33.3% (21/63)	31.4% (11/35)		28.6% (18/63)	57.1% (20/35)		41.3% (26/63)	88.6% (31/35)	
Presence of foci	Absent	30.2% (19/63)	48.6% (17/35)	0.04	90.5% (57/63)	97.1% (34/35)	0.42	74.6% (47/63)	45.7% (16/35)	<0.001
	Hypoechoic foci	1.6% (1/63)	11.4% (4/35)		0% (0/63)	0% (0/35)		11.1% (7/63)	45.7% (16/35)	
	Hyperechoic foci	68.3% (43/63)	40.0% (14/35)		9.5% (6/63)	2.9% (1/35)		14.3% (9/63)	8.6% (3/35)	

Note.- Nonneo: nonneoplastic polyp, Neo: neoplastic polyp.

#### *Step 2: Diagnostic performance of the DL-DSS*

The diagnostic performance of the DL-DSS showed an AUC of 0.92, sensitivity of 26/35 (74.3%), specificity of 58/63 (92.1%), and an overall accuracy of 84/98 (85.7%) (Table 2). One radiologist with 11 years of abdominal ultrasound experience showed better performance than the DL-DSS. However, the system showed a better performance than two reviewers, who had 3 and 5 years of abdominal ultrasound experience. A statistically significant difference was observed between the DL-DSS and reviewer B (p < 0.01).

#### *Step 3: Diagnostic performance of image analysis by three human reviewers with the aid of the DL-DSS*

**Figure 2:**
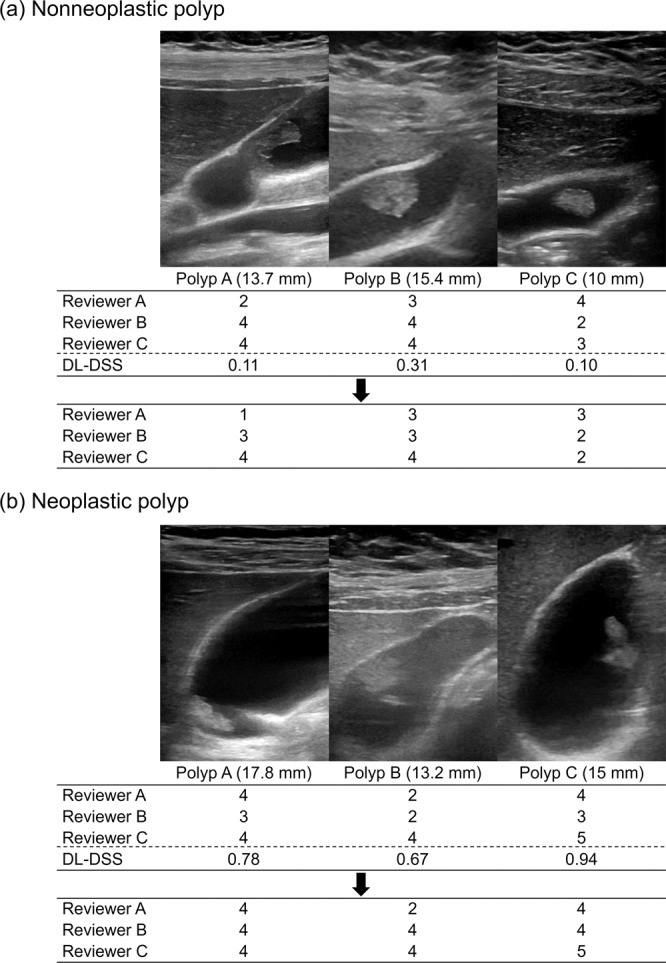
Example cases showing the effectiveness of DL-DSS aided diagnosis. (**a**) Three patients with a nonneoplastic polyp, measured over 10 mm size. Majority of the reviewers regarded these polyps as neoplastic polyp with confidence scale 3 or more. However, patient-level probability value was from 0.1 to 0.3 suggesting nonneoplastic polyp more likely. On the re-evaluation, some of the reviewers downgraded the score. (**b**) Three patients with a neoplastic polyp, measured from 13 to 18 mm size. Some of the reviewers classified these polyps as nonneoplastic polyp with confidence scale 3 or less. On the other hand, patient-level probability value was from 0.7 to 0.9, favoring neoplastic polyp. On the re-evaluation, some of the reviewers upgraded the score.

Table 2 shows that all three reviewers’ diagnostic performances increased when the DL-DSS was added. All three of the reviewers’ AUCs became higher than 0.9. A statistically significant improvement was shown for reviewer B, whose performance increased from 0.78 to 0.91 (p < 0.01). Among the sensitivity, specificity, and accuracy, the specificity showed marked growth in all three reviewers (Fig. 2). In addition, the interobserver agreement improved with the aid of the DL-DSS (ICC from 0.87 to 0.93).

### Diagnostic performance for GB polyps larger than 10 mm

Table 4 summarizes the diagnostic performance of the three human reviewers and the DL-DSS for GB polyps larger than 10 mm. When the GB polyps were larger than 10 mm, the AUC of all experiments became lower. However, the added gain of the DL-DSS became more evident. For the human reviewers, the gap between sensitivity and specificity became more prominent. The sensitivity and specificity were from 69.7 to 100 and from 41.9 to 77.4, respectively. Additionally, the sensitivity and specificity of the DL-DSS were 78.8 and 87.1, respectively. The maximum gap of specificity between human and DL-DSS was exaggerated from 27 to 45.2. The p-values of the comparison between the 1^st^ and 2^nd^ review became more significant (<0.001–0.16).

**Table 4:** Diagnostic Performance of Reviewers and DL-DSS for GB polyps larger than 10 mm.

	AUC	Comparison	Sensitivity	Specificity	Accuracy	F-1 Score
**Step 1: Initial Image Analysis**		Step 1 vs Step 2 (p value)				
Reviewer A	0.92 [0.82–0.97]	0.96	90.9 (30/33) [75.7–98.1]	77.4 (24/31) [58.9–90.4]	89.1 (57/64) [67.6–94.4]	0.857 [0.769–0.895]
Reviewer B	0.68 [0.55–0.79]	<0.001	69.7 (23/33) [51.3–84.4]	54.8 (17/31) [36.0–72.7]	62.5 (40/64) [43.9–78.7]	0.657 [0.530–0.744]
Reviewer C	0.82 [0.70–0.90]	0.04	100 (33/33) [89.4–100.0]	41.9 (13/31) [24.5–60.9]	71.9 (46/64) [58.0–81.1]	0.786 [0.733–0.814]
**Step 2: DL-DSS Alone**						
DL-DSS	0.92 [0.82–0.97]		78.8 (26/33) [61.1–91.0]	87.1 (27/31) [70.2–96.4]	82.8 (53/64) [65.5–93.6]	0.825 [0.705–0.896]
**Step 3: DL-DSS Aided**		Step 1 vs Step 3 (p value)				
Reviewer A	0.94 [0.84–0.98]	0.16	87.9 (29/33) [71.8–96.6]	93.6 (29/31) [78.6–99.2]	90.7 (58/64) [75.1–97.9]	0.906 [0.807–0.953]
Reviewer B	0.89 [0.79–0.96]	<0.001	84.9 (28/33) [68.1–94.9]	87.1 (27/31) [70.2–96.4]	86.0 (55/64) [69.1–95.6]	0.862 [0.756–0.917]
Reviewer C	0.89 [0.79–0.96]	0.08	97.0 (32/33) [84.2–99.9]	54.8 (17/31) [36.0–72.7]	76.6 (49/31) [60.9–86.7]	0.810 [0.743–0.825]

Note.- Data in brackets are 95% CI.

## Discussion

The DL-DSS could be a useful assistant tool for the differential diagnosis of neoplastic GB polyp by decreasing the gap between reviewers with different experience levels and reducing the false positive rate. The initial AUCs for neoplastic GB polyp were 0.94, 0.78, and 0.87. With the DL-DSS assistant, reviewers’ performances improved to 0.95, 0.91, and 0.91. The diagnostic performance of the less-experienced radiologists significantly improved with the aid of the DL-DSS (0.78 to 0.91 p < 0.01 for Reviewer B and 0.87 to 0.91, p = 0.17 for Reviewer C). The ICC improved from 0.87 to 0.93 with the aid of DL-DSS.

Among the important US finings for neoplastic polyps such as a single, larger, and sessile polyp, a larger size was the only independent factor on the multivariable regression. The optimal cutoff dividing the nonneoplastic polyp and neoplastic polyp was 13.1 mm. The importance of the size also had been reported on previous studies. According to Yeh C-N’s reports^19^, a size criteria that was equal to 10 mm or larger was the independent risk factor for neoplastic polyp. Cha BH, *et al*. collected 210 patients who had a GB polyp larger than 10 mm, and found that a size equal to 15 mm or larger was an independent risk factor suggesting neoplastic polyp^20^. Choi TW, *et al*. collected 136 patient’s high-resolution US images and reported that a single polyp and large diameter were the meaningful factors predicting a neoplastic polyp^21^.

The DL-DSS showed a higher specificity than all of the human reviewers, and this characteristic helped in improving the human performance. Although the human reviewers mainly relied on the size of polyp to differentiate neoplastic polyp, there was a substantial overlap zone between nonneoplastic polyps and neoplastic polyps in the size distribution plot. On the subgroup analysis for the large polyp (≥10 mm), the specificity of the human reviewers dropped markedly than the DL-DSS. The human reviewers overcalled the neoplastic polyp when the polyp measured 10 mm or larger, and this trend led to unnecessary cholecystectomies. The DL-DSS could reduce the false positive rate, thereby avoiding unnecessary cholecystectomy.

The DL-DSS could help to improve a less-experienced radiologist’s performance and narrow the gap between reviewers. Nam JG, *et al*. demonstrated that the malignant pulmonary nodule detection deep learning algorithm improved the nodule detection performance of 18 physicians with different experience levels^16^. The performance gap between the thoracic radiologist group and other physicians became lower. Bien N, *et al*. developed and validated the deep learning model to detect a general abnormality, anterior cruciate ligament tear, and meniscal tear on knee MRI data^17^. The model improved the interrater reliability among the 7 general radiologists and 2 orthopedic surgeons. Our study results also showed that the diagnostic performance of a nonexperienced radiologist significantly improved with the aid of the DL-DSS (0.78 to 0.91 p < .01 for Reviewer B and 0.87 to 0.91, p = 0.17 for Reviewer C).

Our study has some limitations. First, this study was only performed in a single center, and only temporally external validation was done. Second, the DL-DSS differentiated between nonneoplastic polyps and neoplastic polyps rather than diagnosing the malignant polyps. Since malignant polys are rare, not enough of a development dataset could be collected. With the multicenter dataset, the system could also learn this function. Third, in this study, we used Inception V3 and did not compare with more recent networks such as DenseNet, NASNet, etc. This is one of limitation of this research and further study is needed to make optimal DL-DSS. Finally, as a retrospective study, this study could not reflect the real clinical setting. A multicenter prospective study is needed.

In conclusion, we developed the DL-DSS, which has higher specificity at differentiating neoplastic polyps on transabdominal US. When it was used as an assistant tool, the gap between the reviewers with different experience levels narrowed, and the false positive rate was reduced, especially for the polyps with a size of 10 mm or larger. The DL-DSS could be used as an assistant tool for the differential diagnosis of neoplastic GB polyps using US, decrease the gap between the reviewers with different experience levels, and reduce the false positive rate, thus avoiding unnecessary cholecystectomies.

## Materials and Methods

This study was approved by the Institutional Review Board of Seoul National University Hospital (IRB No. 1712–007–903). The Institutional Review Board granted a waiver of informed patient consent due to the retrospective nature of our study. All methods were performed in accordance with the relevant guidelines and regulations.

### Patients

**Figure 3:**
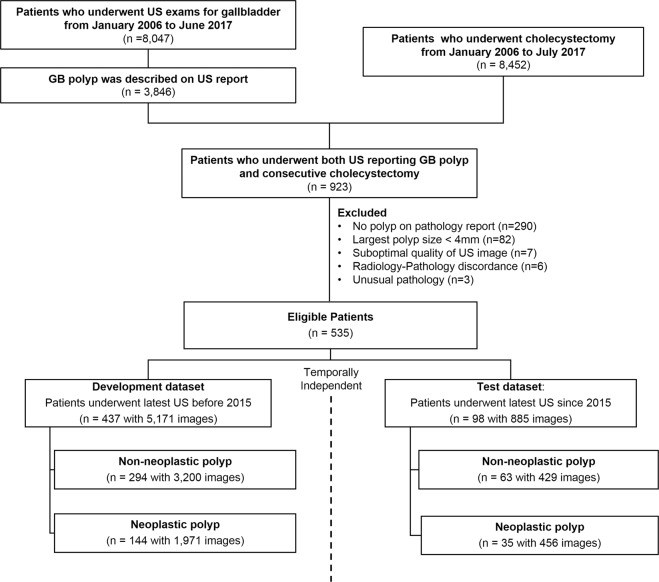
Flow diagrams for the patient selection and dataset division. From our institution’s medical record, we collected 923 patients who examined GB polyp on US and underwent consecutive cholecystectomy. After the exclusion step, we collected total of 535 patients. We divided patients into two temporally independent groups according to study date of their latest US exam.

We reviewed our institution’s medical patient records from 2006 to 2017 and identified 8,452 patients who received cholecystectomy and 8,047 patients who received US for GB. From this data, we collected 923 patients on whom a GB polyp was found through US and received consecutive cholecystectomy. Among them, the following patients were excluded: polyp was not identified on pathologic report (n = 290); polyp smaller than 4 mm in size (n = 82); suboptimal image quality (n = 7); size and location of the polyp was evidently different between US and pathologic report (n = 6); rare pathology such as lymphoma or metastasis (n = 3). Finally, a total of 535 patients were included in our study group, including 357 patients with nonneoplastic polyps and 179 patients with neoplastic polyps. We divided selected patients into two groups to make two temporally independent datasets: the development dataset and test dataset. According to study date of their latest US exam, the patients who received US before January 2015 were considered as the development dataset (n = 437), and the patients who received US from January 2015 were grouped as the test dataset (n = 98). Figure 3 shows the flowchart of this study population.

### US examination

An US examination was performed by the radiologists, who have approximately 10 to 20 years of abdominal ultrasound experience, using an US scanner (LOGIQ 9 or LOGIQ E9, GE Healthcare, Milwaukee, WI, USA). First, the GB was observed grossly by a subcostal or intercostal approach with the convex low-MHz transducer (C1–6 or 4C, GE Healthcare, Milwaukee, WI, USA). Then the high resolution images were achieved by a linear transducer (9L or 7L, GE Healthcare, Milwaukee, WI, USA), which produced a high frequency ultrasound wave with a bandwidth of 2–8 MHz^21,22^.

### Development of DL-DSS

#### *Labeling of dataset*

The images were selected from each patient’s US studies according to the following criteria: only the B-mode images were captured with the linear transducer, the images did not contain any annotation such as size measurement, and the captured polyp should match the pathology report in terms of its size and location. The US image selection and labeling process were performed by two radiologists (Kim JH and Jeong YB, with 23 years and two years of abdominal ultrasound experience, respectively) with consensus. If one patient received serial US follow up, all of the US studies were included when the patient was in the development dataset. By contrast, only the latest US study was included when the patient was in the test dataset. All of the selected images were converted into Portable Network Graphics (PNG) format. Then, all of the images were manually cropped using an in-house program. In every image, we drew a free size square box that contained one polyp and its attachment site. The square box was drawn as small as possible to contain the whole following structures; polyp, stalk, and focal portion of GB wall the polyp attached. The center of the box was located as close as to the polyp’s center. If there were multiple polyps in one image, each meaningful polyp that was 4 mm or larger and matched the pathologic description was cropped separately. We evaluated the size of each polyp simultaneously. We used the size that was measured by the onsite radiologist when it was available. If not, a retrospective size estimation was conducted using the scale bar in the image.

#### *Training for the DL-DSS*

**Figure 4:**
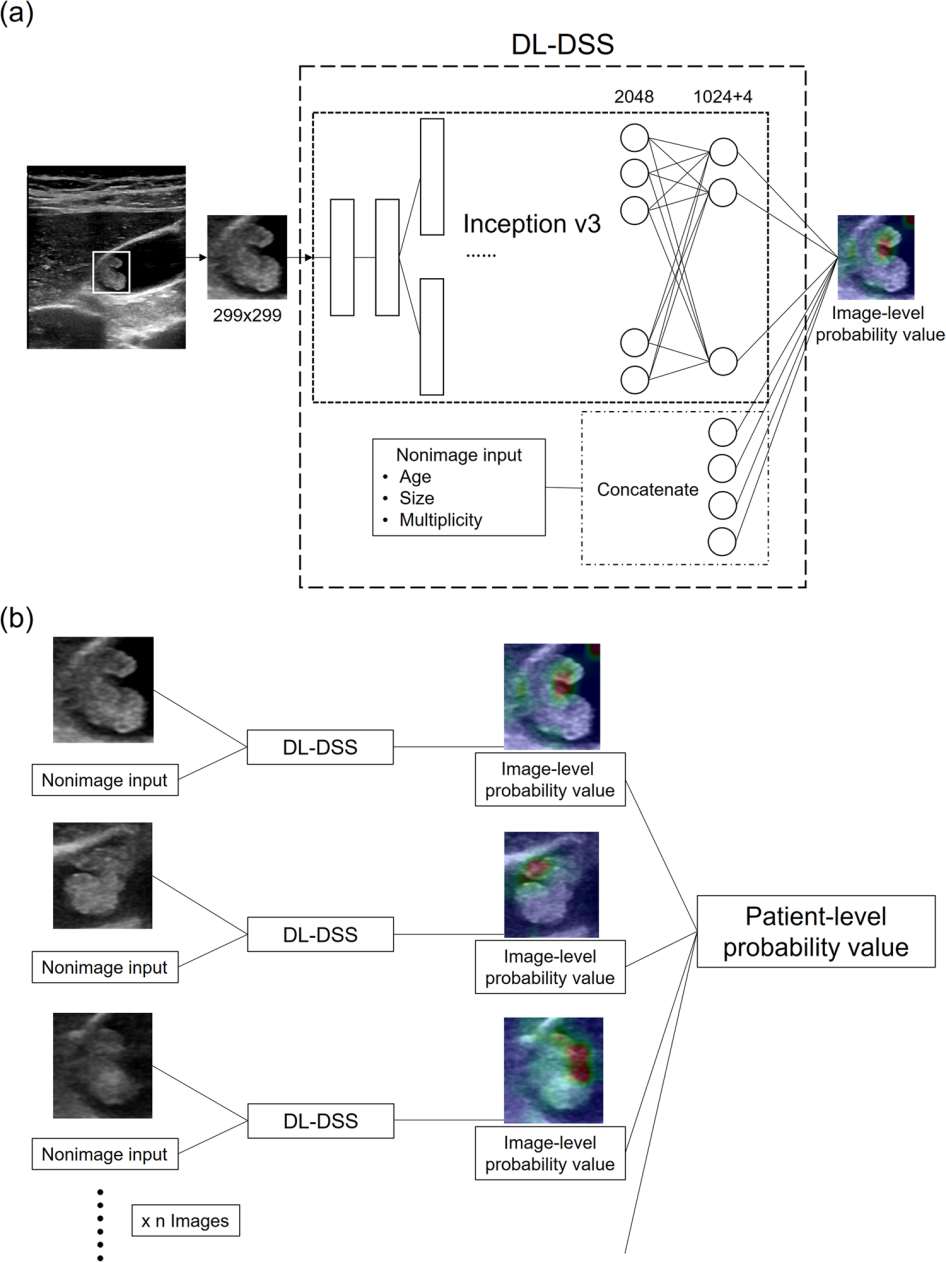
Schematic diagram of DL-DSS. (**a**) We used transfer learning method base on the GoogleNet Inception v3 CNN architecture. All cropped Image were resized into 299 × 299 pixels, and then processed with a pretrained Inception v3 model. Nonimage inputs were concatenated to the last fully connected layer of the network using a late fusion strategy. (**b**) As multiple images were included for one patient, the average of the multiple image-level probability values was used as a patient-level probability value. It is a continuous value between 0 and 1. Zero represents a definite nonneoplastic polyp, and vice versa.

We used the transfer learning method base on the GoogleNet Inception v3 Convolutional Neural Network (CNN) architecture^23,24^. DL-DSS used both cropped image and nonimage information. All of the cropped images were resized into 299 × 299 pixels. Intensity normalization was used as a preprocessing method. Geometric image augmentation techniques such as vertical/horizontal flipping, rotation, and cropping were used to reduce overfitting. Then, the image input was processed with a pretrained Inception v3 model to extract the high-level features. Nonimage inputs, including patient age, size of polyp and polyp multiplicity, were concatenated to the last fully connected layer of the network using a late fusion strategy^25^. In the end, DL-DSS reports a probability value that represents the image-level probability of a neoplastic polyp. A schematic diagram of the DL-DSS is described in Fig. 4.

**Figure 5:**
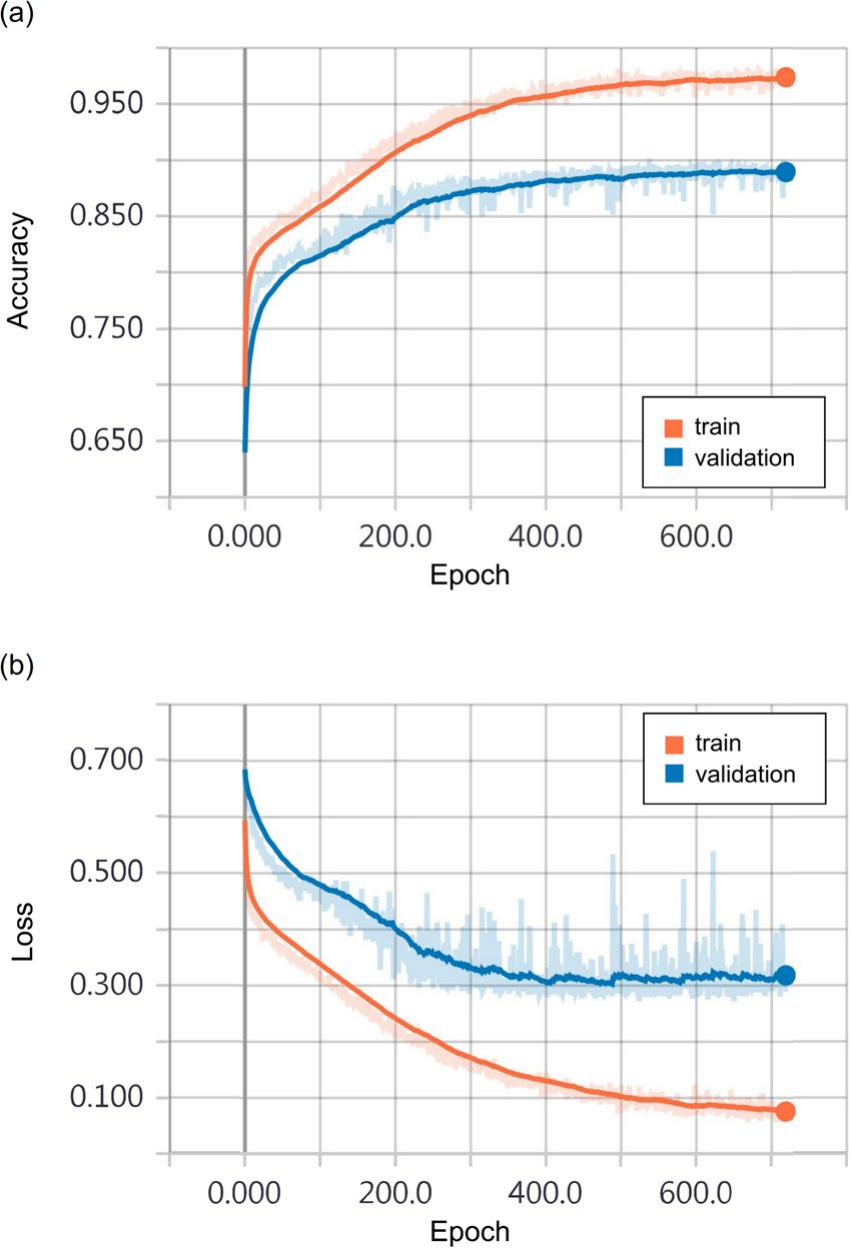
Training curves of DL-DSS. The orange and blue curves represent the (**a**) accuracy and (**b**) loss on the development and test datasets, respectively. We used an early stopping strategy to prevent overfitting, and training was performed up to 400 epochs where validation loss has reached a plateau. Final accuracy was 96.69% and 88.58% in the development set and test set.

The development dataset was randomly divided into an 8:2 ratio and was then used for development (4138 images) and test (1033 images). The training process was done with a learning rate of 0.001 and an RMSprop optimizer. We used an early stopping strategy to prevent overfitting, and training was performed for up to 400 epochs where validation loss has reached a plateau (Fig. 5).

### Clinical validation of the DL-DSS

#### *Step 1: Image analysis by three human reviewers*

Two abdominal radiologists (Reviewers A and B, with 11 and 5 years of abdominal ultrasound experience, respectively) and one trainee radiology resident (Reviewer C with 3 years of abdominal ultrasound experience) independently reviewed the 98 patients’ US exams in the test dataset. They were blinded to the histologic diagnoses. First, they reviewed the following characteristics of polyp: multiplicity (solitary or multiple), size of the largest polyp (mm), overall shape (pedunculated or sessile), surface contour (smooth or lobulated), presence or absence of a vascular core seen on color Doppler sonography, presence of GB stone, internal echogenicity level (hypoechoic or iso- to hyperechoic), internal echogenicity pattern (homogenous or heterogenous) and presence of hyper- or hypoechoic foci. When the patient had multiple polyps, the largest polyp was evaluated. The detail method to evaluate the polyp characteristics was based on the previous researches^21,22^. Second, the radiologists graded the possibility of a neoplastic polyp using a 5-point confidence scale (1: definitely a nonneoplastic polyp; 2: probably a nonneoplastic polyp; 3: borderline; 4: probably a neoplastic polyp; and 5: definitely a neoplastic polyp) for each patient. The grading was based on not only the reviewers own experience, but also the polyp characteristics. All reviewers knew the relationship between the risk of neoplastic polyp and polyp characteristics, based on two previous researches^21,22^.

#### *Step 2: Image analysis by DL-DSS*

A temporally external validation of the DL-DSS was done with the 98 patients in the test set. Unlike human reviewers’ 5-point confident scale, the DL-DSS output the decimal probability value range from 0 to 1 (0: definitely a definite nonneoplastic polyp, and 1: definitely a neoplastic polyp). As multiple images were included for one patient, the average of the multiple image-level probability values was used as a patient-level probability value (Fig. 4). Using the patient-level probability value from each patient, we performed a ROC curve analysis and calculated the sensitivity, specificity, and accuracy of the DL-DSS.

#### *Step 3: Image analysis by three human reviewers with the aid of the DL-DSS*

To evaluate the added value of the DL-DSS aided diagnosis, a second review was conducted by three reviewers after 4 months since the first human review. They were still blinded to the histologic diagnoses. The following DL-DSS analysis data from each patients of test dataset was provide to the reviewers: the number of cropped images analyzed by DL-DSS, patient-level probability value, and standard deviation of multiple image-level probability values. US-images and previous 5-point confidence scale that own rated were also provided to the reviewers. Finally, considering all this information from the DL-DSS, including DL-DSS performance data and DL-DSS analysis data, the reviewers rerated the 5-point confidence scale. We also provided the performance of the DL-DSS on development dataset was introduced, by providing the ROC curve, sensitivity, specificity, and accuracy (with cut-off probability value 0.5).

### Statistical analysis

The population characteristics, such as man to woman ratio, a proportion of nonneoplastic and neoplastic polyps, were compared using a Pearson’s Chi-square test between the development set and test set. Also, patient age and size of polyp were compared using an independent t test. These method were also used to evaluate characteristic differences between the nonneoplastic polyp group and the neoplastic polyp group, in both the development set and test set.

In the step 1, polyp characteristics were compared between the nonneoplastic polyps and neoplastic polyps using a Pearson’s Chi-square test and Fisher’s exact test. The sizes of the polyps were compared using an independent t test. A logistic regression analysis was performed to extract meaningful predictors for neoplastic polyp. After the univariate analysis with the US findings, variables with p values less than 0.05 were chosen for the multivariate analysis. Among three reviewers, the interobserver agreement was calculated with an ICC for each US finding and confidence scale. An ICC was classified into three levels of agreement: 0.59 and less as poor agreement, 0.60–0.79 as moderate agreement, and 0.80–1.00 as good correlation.

In step 1 and step 3, the diagnostic performance evaluation of reviewers was done by a ROC curve analysis, using a 5-point confidence scale. Sensitivity, specificity, accuracy, and F-1 score were calculated with a cut-off confident scale (>3). In step 2, the diagnostic performance evaluation of the DL-DSS was done by a ROC curve analysis, using a patient-level probability value. Sensitivity, specificity, accuracy, and F-1 score were calculated with a cut-off probability value (>0.5). An AUC comparison was performed by using a pairwise comparison ROC curve analysis.

All of the analyses were performed with software (SPSS version 25.0, IBM, Armonk, NY; and MedCalc version 18.2, MedCalc Software, Ostend, Belgium). For all of the tests, a P value less than 0.05 was considered to indicate statistical significance.

## Data Availability

The datasets generated during the current study are not publicly available due to our institutional review board prohibits publication of patient’s personal medical records.
